# Imaging Mass Spectrometry by Matrix-Assisted Laser Desorption/Ionization and Stress-Strain Measurements in Iontophoresis Transepithelial Corneal Collagen Cross-Linking

**DOI:** 10.1155/2014/404587

**Published:** 2014-09-02

**Authors:** Paolo Vinciguerra, Rita Mencucci, Vito Romano, Eberhard Spoerl, Fabrizio I. Camesasca, Eleonora Favuzza, Claudio Azzolini, Rodolfo Mastropasqua, Riccardo Vinciguerra

**Affiliations:** ^1^Humanitas Clinical and Research Center, Via Manzoni 56, Rozzano, Italy; ^2^Department of Surgery and Translational Medicine, Eye Clinic, University of Florence, Florence, Italy; ^3^Department of Ophthalmology, Second University of Naples, Naples, Italy; ^4^University Hospital Carl Gustav Carus, Dresden, Germany; ^5^Department of Surgical and Morphological Sciences, Section of Ophthalmology, School of Medicine, University of Insubria, Via Guicciardini 9, Varese, Italy; ^6^Ophthalmology Unit, Department of Neurological Neuropsychological, Morphological and Movement Sciences, University of Verona, Verona, Italy

## Abstract

*Purpose.* To compare biomechanical effect, riboflavin penetration and distribution in transepithelial corneal collagen cross-linking with iontophoresis (I-CXL), with standard cross linking (S-CXL) and current transepithelial protocol (TE-CXL). *Materials and Methods.* The study was divided into two different sections, considering, respectively, rabbit and human cadaver corneas. In both sections corneas were divided according to imbibition protocols and irradiation power. Imaging mass spectrometry by matrix-assisted laser desorption/ionization (MALDI-IMS) and stress-strain measurements were used. Forty-eight rabbit and twelve human cadaver corneas were evaluated. *Results.* MALDI-IMS showed a deep riboflavin penetration throughout the corneal layers with I-CXL, with a roughly lower concentration in the deepest layers when compared to S-CXL, whereas with TE-CXL penetration was considerably less. In rabbits, there was a significant increase (by 71.9% and *P* = 0.05) in corneal rigidity after I-CXL, when compared to controls. In humans, corneal rigidity increase was not significantly different among the subgroups. *Conclusions.* In rabbits, I-CXL induced a significant increase in corneal stiffness as well as better riboflavin penetration when compared to controls and TE-CXL but not to S-CXL. Stress-strain in human corneas did not show significant differences among techniques, possibly because of the small sample size of groups. In conclusion, I-CXL could be a valid alternative to S-CXL for riboflavin delivery in CXL, preserving the epithelium.

## 1. Introduction

Keratoconus is a slowly progressive, asymmetric, bilateral degenerative corneal disease [1]. The architecture of keratoconic cornea is characterized not only by a different distribution and expression of collagen fibrils, but also by an alteration of interfibrillary distance, therefore reducing corneal stability [2]. Corneal collagen cross-linking (CXL) is presently the only treatment that can relent or arrest progressive ectasia [3–5]. It is based on a photooxidative reaction, catalyzed by riboflavin (vitamin B2), and it induces a biomechanical response that enhances corneal stiffness and blocks the progression of the disease [3, 5–7]. This bears consequences on corneal biomechanics, with visual acuity, morphological and functional indices improving up to 48 months postoperatively and possibly more [3–5]. Standard CXL (S-CXL) technique involves the removal of corneal epithelium to allow penetration of riboflavin. Epithelial debridement causes pain [8] and an increased risk of corneal infection [9] in the immediate postoperative period, as well as visual loss during the first months after treatment [4]. In the attempt to avoid these disadvantages, transepithelial CXL (TE-CXL) technique was introduced, with a protocol based on a specially formulated riboflavin solution, Ricrolin TE (Sooft, Montegiorgio, FM, Italy), featuring two enhancers, trometamol and sodium EDTA, in order to improve stromal penetration.

There are several long-term follow-up studies [3, 4, 6, 7, 10, 11], all adopting the standard technique, while there are only few reports on long-term results of TE-CXL, with controversial outcomes [12–16].

The use of enhancers is not the only way to increase riboflavin penetration through an intact epithelium. Another possibility is iontophoresis, a technique in which the drug is applied with an electrode of the same drug charge. A ground electrode, of opposite charge, is placed elsewhere on the body to complete the electric circuit. The drug serves as a conductor of the current through the tissue. Riboflavin penetration and distribution in the cornea using this technique remains controversial [17, 18].

The aim of this study was to evaluate corneal iontophoresis as a possible alternative to riboflavin corneal stromal impregnation without removing the epithelium, assessing riboflavin corneal penetration and distribution along with the best UV irradiation power to obtain adequate outcomes.

Initially, we evaluated riboflavin corneal penetration and distribution in rabbit eyes following different soaking protocols: standard, transepithelial, and iontophoresis-assisted corneal imbibition. Then we evaluated the biomechanical effect of iontophoresis corneal collagen cross-linking (I-CXL) with stress-strain measurements in rabbits and ex vivo human corneas.

## 2. Material and Methods

The study was divided in two different sections, one on pigmented rabbits (strain GD79b) and one on human cadaver corneas. One group of rabbit corneas was utilized to evaluate riboflavin distribution in the tissue with imaging mass spectrometry by matrix-assisted laser desorption/ionization (MALDI-IMS), whereas a second one, as well as the human corneas, was used for the evaluation of stress-strain.

The experiments were performed in the laboratory of Humanitas Clinical and Research Center, Rozzano, and in the Eye Clinic, University of Florence, Italy while the biomechanical assay was accomplished at the University Hospital Carl Gustav Carus, Dresden, Germany. Different riboflavin solutions and cross-linking techniques were evaluated.

We hereafter describe the different riboflavin solutions and cross-linking techniques adopted.

### 2.1. Riboflavin Solutions

Riboflavin preparations used were as follows: Riboflavin 0.1% with dextran T500 20% (Ricrolin, Sooft, Montegiorgio, FM, Italy); Riboflavin 0.1% with dextran T500 15%, plus EDTA and trometamol enhancers (Ricrolin TE, Sooft, Montegiorgio, FM, Italy); Riboflavin 0.1%, dextran free, with NaCl, plus EDTA and trometamol enhancers (Ricrolin preparation A, Sooft, Montegiorgio, FM, Italy); Riboflavin 0.1%, specifically formulated for iontophoresis, dextran free, without NaCl, plus EDTA and trometamol enhancers (Ricrolin +, Sooft, Montegiorgio, FM, Italy).

### 2.2. Standard Cross-Linking Technique

#### 2.2.1. Imbibition

S-CXL was done according to the Dresden protocol [5]. The corneal epithelium was mechanically removed in a central 9-mm diameter area. A solution of riboflavin 0.1% and dextran 20% (Ricrolin Sooft, Montegiorgio, Italy) was instilled every minute for 30 minutes to fully irrigate the cornea. 

#### 2.2.2. Irradiation

A 7.5 mm diameter of the central cornea was then irradiated with an irradiance of 3 mW/cm^2^ (VEGA CBM x-linker, C.S.O, Florence, Italy) for 30 minutes. The solution was instilled every 5 minutes during the UVA treatment.

### 2.3. Transepithelial Cross-Linking Technique

#### 2.3.1. Imbibition

In TE-CXL, Ricrolin TE was applied on the corneas every minute for 30 minutes.

#### 2.3.2. Irradiation

The central cornea was then similarly irradiated with an irradiance of 3 mW/cm^2^ (VEGA CBM x-linker, C.S.O, Florence, Italy) for 30 minutes. The solution was instilled every 5 minutes during the UVA treatment. 

### 2.4. Iontophoresis Cross-Linking Technique

#### 2.4.1. Imbibition

Soaking time with iontophoresis in rabbit and human corneas was performed without the removal of the corneal epithelium, using the same iontophoretic device. This consists of two disposable components: an ocular applicator and a return electrode, both connected to a reusable generator. In rabbit corneas the ocular applicator consisted of a 10 mm wide—4.5 mm high round polycarbonate reservoir, filled with medical foam PUR (Advanced Medical Solutions BV, Etten-Leur, Netherlands) and a stainless steel electrode connected to the generator (cathode). The return electrode was a 25 G intradermic needle, inserted in the rabbit's neck (front side) and connected with a crocodile clip and lead to the generator (anode). A constant current generator (I-ON XL, Sooft, Montegiorgio, FM, Italy) was used, with a setting range of 1 mA for 5 minutes. The voltage applied during the study was measured with a multimeter.

**Figure 1:**
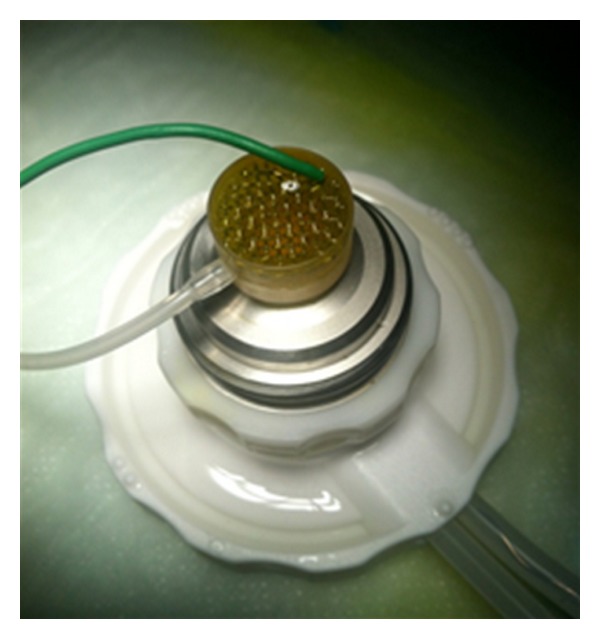
Iontophoresis applicator for human donor corneas.

In human corneas, a 8 mm-wide iontophoretic application device was placed on the corneal surface using an annular suction ring. The device was filled with approximately 0.5 mL solution from the open proximal side, until the electrode (stainless steel mesh) was covered. The device was connected to the constant current generator for 5 minutes (I-ON XL, Sooft, Montegiorgio, FM, Italy) set at 1 mA (the total dose of 5 mA/5 min was monitored by the generator). Human corneas were placed on an artificial anterior chamber (Moria USA, Doylestown, PA) and the return electrode was a stainless steel wire inserted into one of the pressure tubes, underneath the corneas, in the artificial anterior chamber. The two tubes were connected to a perfusion line allowing BSS circulation during the study (Figure 1). Attention was paid to eliminate any air bubble in the circuit. 

#### 2.4.2. Irradiation

Irradiation power in rabbits and human I-CXL corneas was either 3 mW for 30 minutes or 10 mW for 9 minutes (VEGA CBM x-linker, C.S.O, Florence, Italy) according to the group of randomization.

#### 2.4.3. Rabbit Corneas

Twenty-four pigmented rabbits (twelve for the MALDI-IMS experiment and twelve for stress-strain measurements), aged 2 to 3 months, with a weight ranging from 2.0 kg to 2.5 kg, were used. All animals were healthy and free of ocular disease. Rabbits were handled according to the European Commission and the Association for Research in Vision and Ophthalmology (ARVO) Statement for the Use of Animals in Ophthalmic and Vision Research. Rabbits were anesthetized with a mixture of ketamine and xylazine hydrochloride. After the treatment, the animals were euthanized by an intravenous injection of overdosed pentobarbital and all efforts were made to minimize suffering.

In the MALDI-IMS study all rabbits eyes (24 eyes) were used for imbibition assessment, conversely in the stress-strain study (24 eyes) one eye of each rabbit was treated as randomized, the other eye was included in the sham group.

### 2.5. Imaging Mass Spectrometry by Matrix-Assisted Laser Desorption/Ionization (MALDI-IMS) Study

The experiments were performed in the Eye Clinic, University of Florence, Italy. In order to study tissue distribution of riboflavin, IMS was used. IMS is a relatively new technique that allows detecting the presence of a substance in a tissue without labelling it [19]. In particular, MALDI-IMS is able to identify a compound or its metabolites by detecting specific peaks in their mass spectra with a histologic resolution of about 50 *μ*m. It has recently been used for pharmacokinetics studies of drug distribution in the eye [20].

MALDI ion source is formed by a nitrogen laser with UV emission. The laser gives energy to the matrix crystals causing desorption and ionization. Matrix and analytes (in ionic form) enter the mass spectrometer analyser and are detected.

Twenty-four rabbit eyes were evaluated. They were divided into three groups composed of eight eyes of four rabbits each, according to the different imbibition protocols: S-CXL, TE-CXL, and I-CXL. In order to evaluate riboflavin corneal penetration and distribution following different soaking protocols and differently from those involved in the stress-strain study, these eyes were not irradiated after the imbibition procedure.

**Figure 2:**
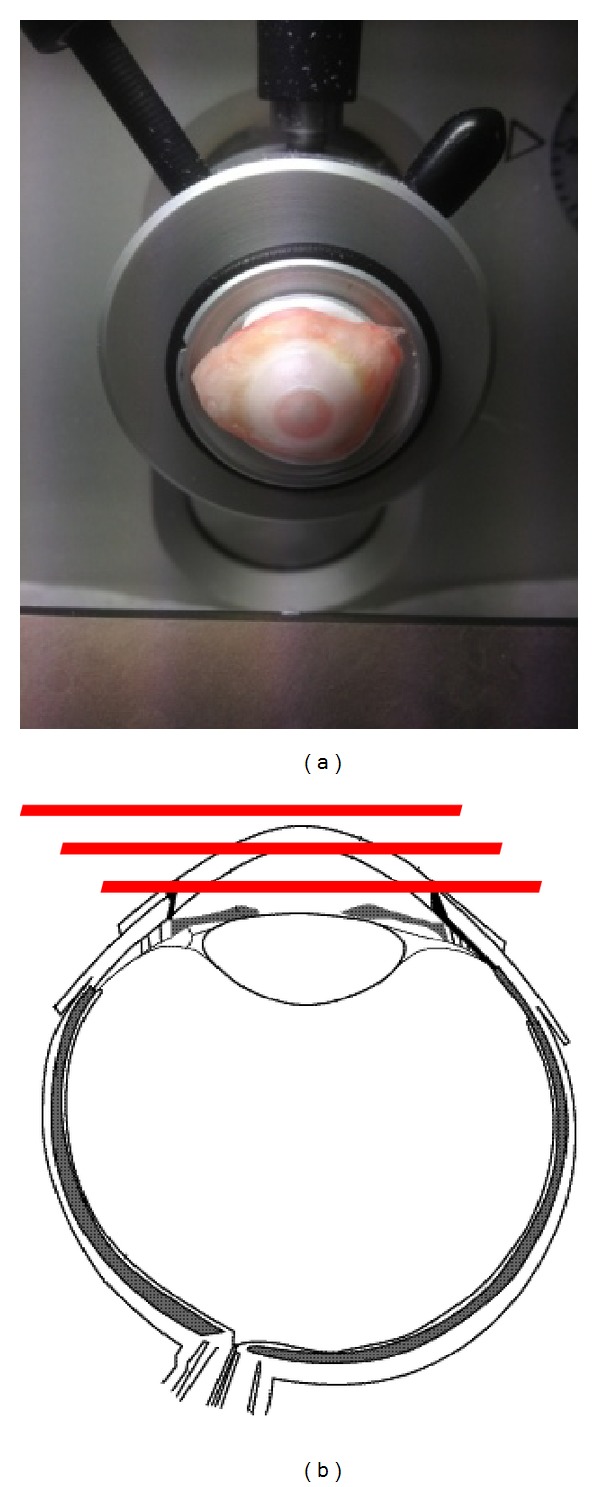
Corneal section preparation for MALDI-IMS. Corneas of enucleated globes were cut in frontal sections (slice thickness about 20 *μ*m) using a cryostat-microtome.

**Figure 3:**
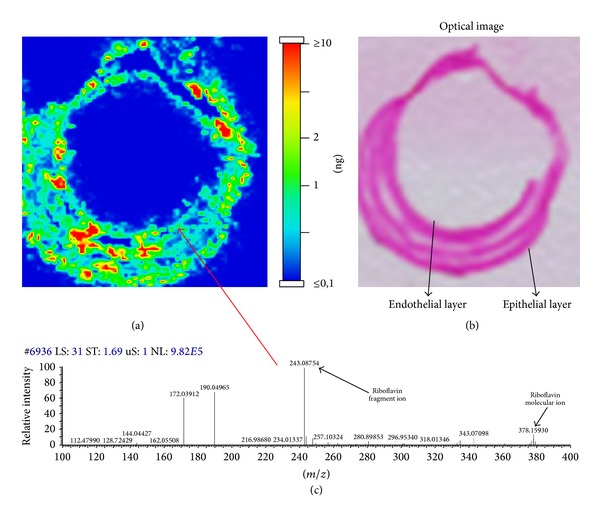
Example of a MALDI-MS imaging of a corneal section soaked with riboflavin, with the colour scale (a). Haematoxylin-eosin stained samples after MALDI-MS imaging and matrix removal (b). Example of MALDI-IMS spectrum of riboflavin that describe one point of the plot (c).

After sacrifice, whole ocular globes were dissected and frozen with isopentane vapor at −80°C. Corneas of enucleated globes were cut in frontal sections (slice thickness about 20 *μ*m) using a cryostat-microtome (Figure 2). Cornea sections were placed on a histology glass and dried under low vacuum inside a desiccator for 2 hours and then coated with a MALDI matrix: α-cyano-hydroxycinnamic acid 10 g/l solution in 50% acetonitrile, plus 0,1% TFA and equimolar quantity of aniline. Matrix coating was done by an automated spraying device (ImagePrep, Bruker Daltonics, Billerica, MA, USA). Every sample was then analysed using a high resolution hybrid MALDI-mass spectrometer. Sample analyses were conducted in raster mode at 50 *μ*m raster size, collecting 20 laser shots at 5 *μ*j laser energy, allowing the estimation of riboflavin localization and distribution inside the corneal layers. Riboflavin fragment ion at* m/z* 243.087 was used for detection into a tandem mass (product ion scan) experiment with 60.000 resolution power. The image was plotted into a colour scale related to the estimated riboflavin quantity for a single voxel (volume of approximately 5 × 10^−5 ^mm^3^) and it represents the riboflavin fragment ion distribution inside this tissue section (Figure 2(a)). The intensity of the ion signal (correlated with the colour scale) is proportional to the riboflavin amount in that given point. For the semiquantitative estimation of riboflavin we used a control tissue, spotted with riboflavin standard solution, running an external calibration curve. Every sample of every group was repeated on at least 5 tissue sections deriving from the same animals (technical replicates). The value presented was averaged on these replicates. Every point of the plot is described by one tandem mass spectrum of the riboflavin (Figure 3(c)).

After IMS analysis, samples were washed with ethanol for matrix removal and prepared for haematoxylin-eosin staining following a standard staining protocol (Figure 3(b)).

Haematoxylin-eosin preparations permit to have an overview of the histological structure of the tissue and to visually locate the topographical distribution of the substance, overlapping it with the IMS images.

### 2.6. Biomechanical Study

The biomechanical study was divided in two different sections: a study on pigmented rabbits (twenty-four eyes) and one on human cadaver corneas (twelve). The experiments were performed in the laboratory of Humanitas Clinical and Research Center, Rozzano, while the biomechanical assay was performed at the University Hospital Carl Gustav Carus, Dresden, Germany.

#### 2.6.1. Rabbit Corneas

Twelve pigmented rabbits were studied following the same procedure and handling specified above.

They were randomly divided into four groups of three animals each. The groups differed in type of riboflavin used and soaking time: TE-CXL, iontophoresis imbibition with Ricrolin TE, iontophoresis imbibition with Ricrolin prep A, and iontophoresis imbibition with Ricrolin + (see also Table 1). Randomization in the treatment groups was done using Excel software (Microsoft Office 2007). One eye of each rabbit was treated as randomized; the other eye was included in the sham group.

All treated eyes were irradiated with a power of 3 mW for 30 minutes.

Immediately after sacrifice, both eyes were quickly and carefully sampled, weighed, and stored in a wet chamber at 4°C (histological screw cap container filled with cotton soaked with 0,9% saline), until shipment for assay. The sampling time was equal for each rabbit (10 minutes). Central corneal thickness (CCT) was measured with a pachymeter (Pach-Pen XL; Mentor, Norwell, MA, USA) and mean CCT was 708.6 ± 52.9 *μ*m. The corneoscleral ring was removed and the cornea was cut into 2 equal strips 5 mm wide and 7 mm long including 1 mm of sclera on both ends.

#### 2.6.2. Human Corneas

Twelve single human corneal-scleral discs, and qualified for research use, were obtained by the biorepository of The Veneto Eye Bank Foundation, Venice (Italy). Before recovery, a written consent from donor's relatives was obtained, in order to get permission for surgical and alternative uses (i.e., education, training, and research purposes).

Corneas were collected after a mean postmortem interval of 6.3 hours (min 1.95, max 9.25 hours), and deemed unsuitable for transplantation because of donor contraindications, other than serology, or stromal abnormalities. These tissues were evaluated, stored in culture according to conventional eye banking procedures [21], and used for our protocols after a mean storage time of 120.4 hours (min 96, max 135.5 hours). All corneas (mean donor's age 63.1, min 43.2, max 73.5 years) displayed a healthy, uninterrupted epithelium. 24 hours before use, tissues were transferred in culture medium +6% dextran, according to conventional organ culture technique, to allow deturgescence. Central endothelial cell density and viability (tripan blu staining) were measured before use. The mean endothelial density was 2400 cells/mm^2^ (min 2200, max 2800 cells/mm^2^), with no evidence of cell mortality.

To reduce the variances in the stress-strain measurements due to different postmortem times and degrees of autolysis, the human corneas were uniformly divided into groups, taking account of the age of the donor values of post mortem interval and mean storage time.

The human corneas were randomly divided into four groups distinguished by method of impregnation and irradiation power, as reported in Table 2. Group A was the S-CXL treatment which entails epithelial debridement, passive soaking, and 3 mW UV-A power for 30 minutes; group B comprised 3 corneas treated with TE-CXL with passive transepithelial soaking and 3 mW UV-A power for 30 minutes; groups C and D were both impregnated with iontophoresis in 5 minutes, however group C was then irradiated with a power of 3 mW for 30 minutes whereas group D with 10 mW for 9 minutes. The randomization in two different irradiation powers for I-CXL was to evaluate if, giving the different impregnation method (I-CXL), the irradiance power had any influence in the results. Corneoscleral discs were gently grasped by the scleral rim and carefully mounted on a perfused artificial anterior chamber (Figure 1) (Moria USA, Doylestown, PA), with the endothelial side down. Once the disc was properly mounted, the epithelium was evaluated under microscope and CCT was determined using an ultrasound pachymeter (SP-2000; Tomey, Erlangen, Germany), showing a mean value of 572.6 ± 71.9 *μ*m. Following treatment, each donor tissue was cut into two equal strips 4 mm wide and 14 mm long including 1 mm of sclera on both ends.

### 2.7. Static Stress-Strain Measurements

Static stress-strain measurements of the corneas were performed using a microcomputer-controlled biomaterial tester (Minimat, Rheometric Scientific GmbH) with a prestress of 5 × 10^3^ Pa in the human corneas and 10 × 10^3^ Pa in the rabbit corneas (1 Pa = 1 N/m^2^). Vertical strips were clamped in the stress-strain device. The distance of the clamps was 7 mm, the load was 5 N, and the preload was 20 mN in the rabbit corneas, while in the human corneas the load was 5 N and the preload was 10 mN. The stress-stain curves were fitted with an exponential function *σ* = *A*exp⁡(*B* × *ε*) using the SPSS-calculation program (SPSS GmbH Software, Munich) and the Young's modulus (relation between tangential force and cross-sectional area) was calculated for 4%, 6%, 8%, and 10% strains as the gradient of the stress-strain graph.

### 2.8. Statistical Evaluation

Statistical analysis was performed using the STATA statistics software version 11.0 (STATA, Texas, USA). Data are described by mean and standard deviation. To test whether more than two independent samples originate from the same distribution we used a nonparametric method, the Kruskal-Wallis one-way analysis of variance by ranks. The Mann-Whitney test for unpaired data was applied to assess the significance of differences between control and treated data from the rabbits, using the same level of significance (*P* ≤ 0.05) in all cases.

## 3. Results

### 3.1. MALDI-IMS Riboflavin Penetration

**Figure 4:**
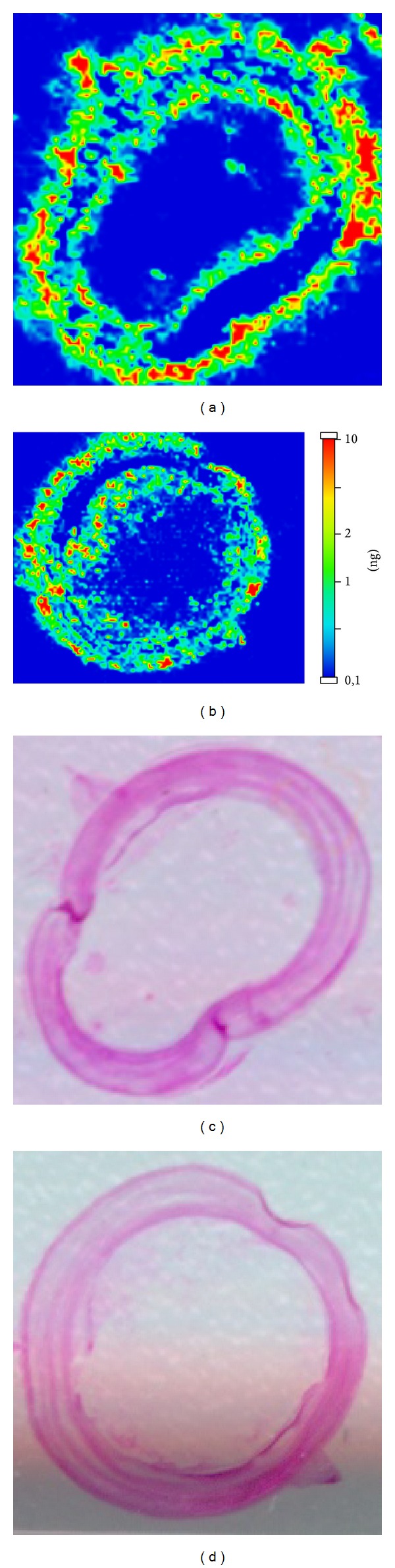
Standard group: MALDI-MS Imaging (a and b) and haematoxylin-eosin staining (c and d) of two corneal samples of two different eyes.

In the standard group, riboflavin was distributed throughout the cornea. Hot colour spots were detected in all corneal layers in depth and up to the limbus (Figure 4).

**Figure 5:**
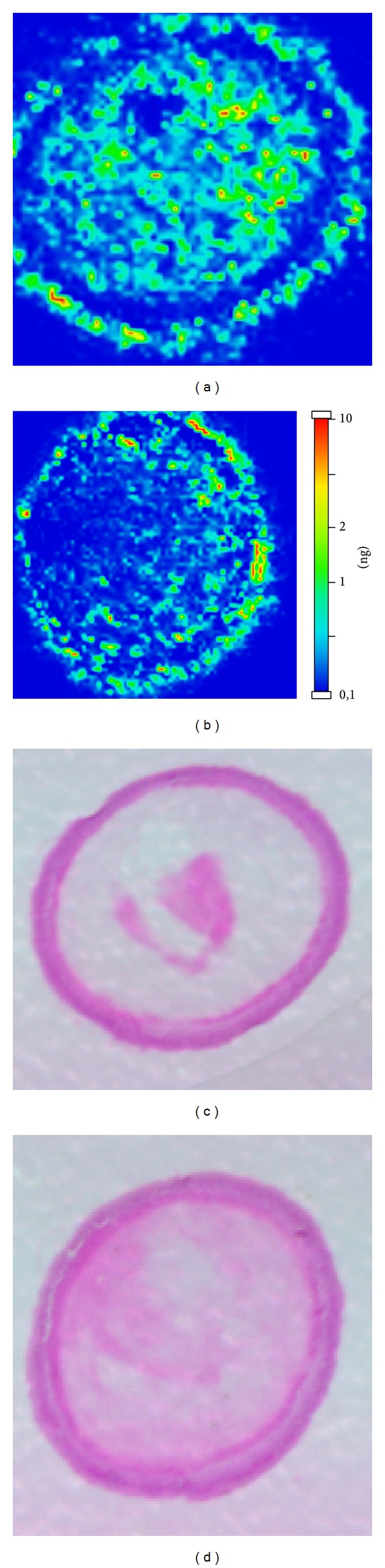
Transepithelial group: MALDI-MS imaging (a and b) and haematoxylin-eosin staining (c and d) of two corneal samples of two different eyes.

Conversely, in the transepithelial group the penetration of riboflavin was reduced in comparison with the standard group of almost 20%. Only faint hot spots can be detected (Figure 5).

**Figure 6:**
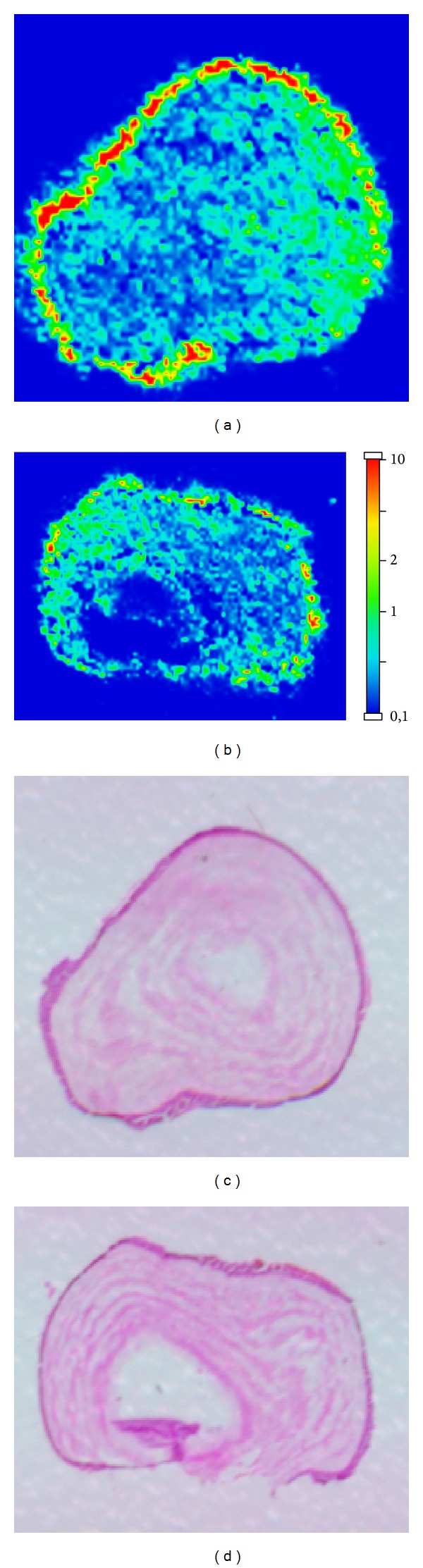
Iontophoresis group: MALDI-MS imaging (a and b) and haematoxylin-eosin staining (c and d) of two corneal samples of two different eyes.

Riboflavin in the iontophoresis group was distributed throughout the corneal layers, in depth, and up to the limbus, with a slightly lower concentration in the deepest layers compared to the standard group (Figure 6).

### 3.2. Biomechanical Essay

#### 3.2.1. Stress-Strain Curve

**Figure 7:**
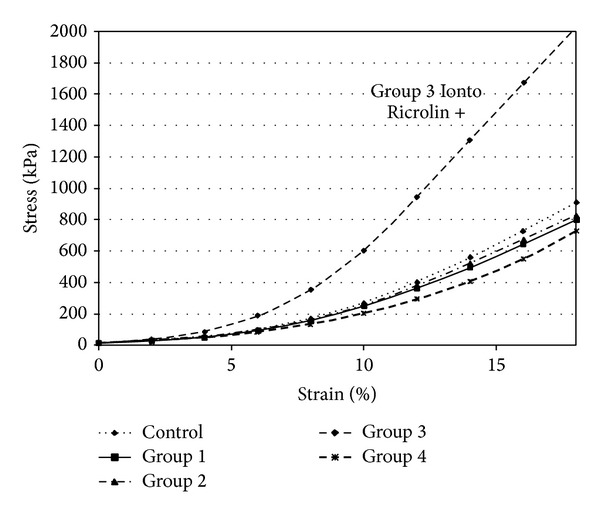
Stress-strain measurements of rabbit corneas.

**Figure 8:**
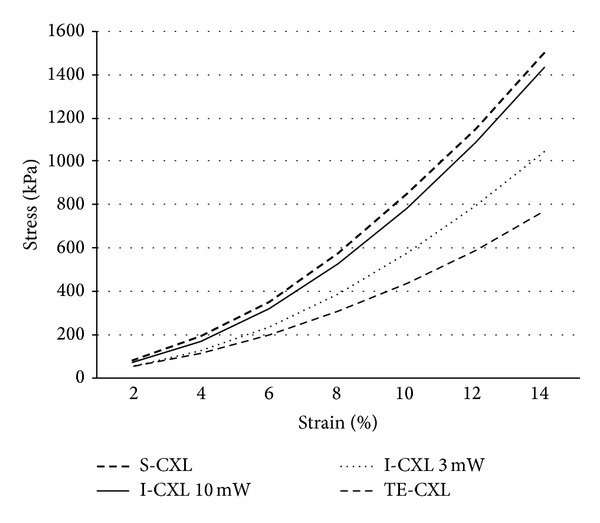
Stress-strain measurements of human corneas.

The stress-strain curves in both experiments presented the typical exponential increase of a bioviscoelastic solid (Figures 7 and 8).

In rabbit corneas, there was no statistically significant difference between CCT between treated and untreated eyes in the four subgroups (*P* = 0.8, *P* = 0.6, *P* = 0.5, and *P* = 0.4). Stress-strain results of the different groups are summarized in Table 3. In Group 3 (I-CXL) the stress using 10% strain was 603,3 ± 316,7 × 10^3^ Pa in the treated corneas and 260,7 ± 40,5 × 10^3^ Pa in the untreated corneas, corresponding to a 71.9% increase (Figure 7). The difference was statistically significant (*P* = 0.05). 

In human corneas, there was no statistically significant difference between CCT of several subgroups (*P* = 0.06). The stress using 4%, 6%, 8%, and 10% strains was not significantly different among subgroups (*P* = 0.2, *P* = 0.2, *P* = 0.1, and *P* = 0.09, resp.), but there was a tendency to a better result with S-CXL. However, one cornea of this group showed an abnormally high result. Stress values with a 10% strain are summarized in Table 4.

#### 3.2.2. Young's Modulus

To calculate Young's modulus, the stress-strain values were fitted with an exponential function *σ* = *A*exp⁡(*B* × *ε*).

**Figure 9:**
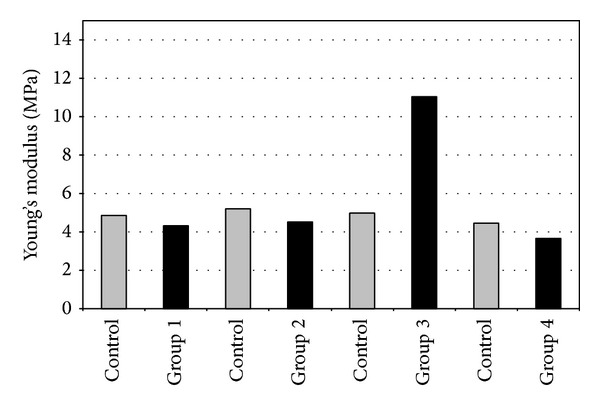
Young's modulus at 10% strain of rabbit corneas.

In rabbit corneas, in the group 3 at 10% strain, Young's modulus was 4,9 × 10^6^ Pa in the untreated eyes and was 11,0 × 10^6^ Pa in the treated eyes, with an increase factor of 0.8 (Figure 9).

**Figure 10:**
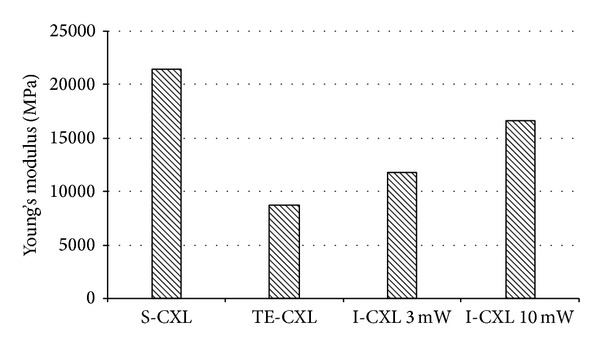
Young's modulus at 10% strain of human corneas.

In human corneas, stress-strain measurements showed an increase in corneal rigidity after CXL in the group treated with S-CXL when compared to other groups. This was shown by a rise in strain and in Young's modulus calculated at 10% strain (Figure 10). None of these differences were statistically significant, possibly because of the small sample size of each group.

## 4. Discussion

Corneal collagen cross-linking is a photochemical reaction aimed at increasing corneal rigidity via the formation intrafibrillar and interfibrillar covalent bonds. To achieve adequate riboflavin penetration in the corneal stroma, the standard cross-linking protocol includes epithelial removal. This induces discomfort [8], temporary vision reduction [4], and risk of infection [9]. In the last years, a cross-linking procedure warranting epithelial integrity but retaining maximal efficacy has been persistently sought for. Nevertheless results of TE-CXL are controversial, with evidences showing that treated patients continue to progress [13], while others show good results [14, 22, 23].

Both corneal epithelium blocking UV penetration and blockage of riboflavin penetration may be important factors for the reduced effect of TE-CXL [24, 25]. For years, the epithelium has been considered a physiological barrier to UV light [24, 25]. Nevertheless, as reported by Kolozsvári et al, epithelium and Bowman's layer mostly absorb UV-B light (up to 300 nm), while UV-A light is not absorbed [26]. Bottós et al. explained the reduced efficacy of TE-CXL with respect to S-CXL, with the hypothesis that the corneal epithelium, while not limiting UVA transmittance, reduces riboflavin penetration [27]. 

If corneal iontophoresis could increase photosensitizer penetration in the cornea, it could overcome the problem. In addition, riboflavin is theoretically a good candidate for ocular iontophoresis due to its negatively charged structure and low molecular weight.

In this study, we compared the changes induced by different CXL techniques in terms of tissue penetration and distribution of riboflavin, as well as of stress-strain. A secondary goal of our study was to evaluate if I-CXL is capable of inducing a good penetration of riboflavin and increase corneal rigidity, differently from TE-CXL.

The hystorical and most accepted approach for evaluating cross-linking efficacy in changing corneal elastic properties is measuring static stress-strain [25, 28]. Several reports in literature showed that S-CXL is able to induce significant increase in corneal stress-strain properties [25, 28–30], while there are only two studies measuring stress-strain after TE-CXL [25, 31]. Wollensak and Iomdina showed that TE-CXL does not induce any significant change in corneal rigidity [25], while Tao et al. reported the opposite [31].

Our biomechanical essay on rabbit corneas showed that I-CXL induces a significant increase in stress-strain when compared to untreated group. The best riboflavin solution for iontophoresis seemed to be the dextran and NaCl-free, with low osmolarity and the addition of enhancers, EDTA, and trometamol. Our findings are partly in agreement with those of Cassagne et al., which showed a significant increase in both stress-strain measurements and Young's modulus in I-CXL-treated rabbit corneas when compared to controls [17].

Rabbit corneas have been used in TE-CXL experiments because the rabbit epithelium is histologically quite similar to that of humans, and therefore recommended by past researchers as a reasonable approximation to clinical reality, avoiding the use of valuable nonhuman, primate research animals [32]. However, it is well known that rabbit corneas lack Bowman's membrane [32] and feature an epithelium which is centrally thicker than at the limbus [33], exactly the opposite of human beings [34]. For this reason, we decided to investigate the effect of I-CXL also in ex vivo human corneas. We did not observe a statistically significant difference between stress-strain values and Young's modulus among the different subgroups. Nevertheless, even if not significantly different, S-CXL treatment with epithelial removal and 10 mW I-CXL presented a tendency to better results when compared to TE-CXL and 3 mW I-CXL.

The increase in stress-strain measurement between TE-CXL, S-CXL, and I-CXL observed in our study differs from what was published by Wollensak et al. [28], who found an increase in biomechanical rigidity by a factor of 4.5. Possible reasons for this difference may be related to specimen preparation. Wollensak et al. treated human corneas within 1 to 2 hours after enucleation: fresh tissues could thus be the reason for their better results. In addition, even if in our study human corneas were grouped uniformly according to the age of donor and the time between death and sampling, variance among stress-strain measurements was noticed. This bias may be one limit of our study.

The second section of our study used MALDI-IMS. The combination of these two techniques in a single experiment offers a unique opportunity to understand the molecular arrangement of any tissue. Compared to the traditional biochemical procedures, based on antibodies or radiolabelling, and limited by the specificity of the used labels and by the number of compounds that can be studied at the same time, IMS recovers the sample molecular content without the need of any a-priori knowledge of the compound to be detected [35]. Another advantage of MALDI-MS imaging is the high sensitivity and specificity of the analysis. Even if this technique allows only a semiquantitative (not numerical) estimation of the concentration of a substance, it is able to give a vivid and clear spatial representation of the penetration and distribution of the compound in a tissue.

In the MALDI-IMS experiment we found different corneal penetrations and distributions of riboflavin solutions currently used in three cross-linking protocols: standard, transepithelial, and iontophoresis. In our study, corneas soaked with iontophoresis showed the presence of riboflavin in all corneal layers, even if with a lower concentration in the deeper stroma when compared with S-CXL protocol. The TE technique samples showed the lowest riboflavin concentration of all groups. Although every semiquantification value has variability between 20% and 30%, typical of IMS-imaging technique, the results of this semiquantitative analysis showed that the concentration of riboflavin among the three procedures was roughly different. Giving the semiquantitative method of analysis IMS should not be compared with the high-performance liquid chromatography (HPLC) analysis showed in other reports [17, 36] which are able to provide reliable concentrations. However, differently from HPLC, in MALDI-IMS the tissue is not homogenized but analysed in whole sections, so it is able to give a vivid and clear spatial representation of the penetration and distribution of the compound in a tissue.

Our findings are in agreement with the literature, even if with a different, semiquantitative technique, showing that iontophoresis imbibition is able to increase the stromal amount of riboflavin when compared to usual transepithelial administration (TE-CXL) [17, 36]. Nevertheless, it reached a lower concentration when compared to conventional, epi-off protocol [17, 36].

To summarize, the aim of transepithelial corneal collagen cross-linking is to reduce the risk of infections, allow faster vision recovery, and decrease treatment time, all without reducing the efficacy of the procedure. We observed that iontophoresis induced acceptable penetration of riboflavin in all corneal layers, which is the basis for an efficient cross-linking [27], even with an intact epithelium. The effective presence of riboflavin together with UV-A produced, as measured in our stress-strain measurements, a significant increase in corneal stiffness in the I-CXL group compared to controls, therefore partly confirming previous report [17].

Nevertheless, riboflavin concentration and stress-strain measurements after I-CXL were inferior to those obtained with the S-CXL. These findings are again in accordance with recent studies [17, 36]. A possible explanation of the reduced effect of I-CXL is that an intact epithelium soaked with riboflavin may partially arrest UV-A light. However, Zhang et al. showed that the epithelial cells are not enriched with riboflavin [37]. For that reason, only a small part of the UV light should be absorbed by the epithelium, approximately 15–20% [38, 39].

Furthermore, since the energy dose of I-CXL is the same as S-CXL protocol (5.4 J/cm^2^), further studies are required to understand if more irradiance power is necessary to reach the standard protocol biomechanical effect, as well as if this lower stiffening effect may however be enough to stabilize an ectatic cornea.

In conclusion, even if more studies are needed to evaluate safety and efficacy, corneal cross-linking with iontophoresis is potentially a valid alternative to standard cross-linking in improving corneal biomechanical properties and reducing postoperative patient pain, risk of infection, and treatment time.

## Figures and Tables

**Table 1:** Allocation of rabbits in the treatment groups, each rabbit showed right eye treated and left eye untreated.

	Group 1	Group 2	Group 3	Group 4
Number of corneas untreated	3	3	3	3
Number of corneas treated	3	3	3	3
Riboflavin	Ricrolin TE	Ricrolin Prep A	Ricrolin +	Ricrolin TE
Epithelium debridement	NO	NO	NO	NO
Soaking time (minutes)	5	5	5	30
Iontophoresis	YES	YES	YES	NO
Irradiation power (mW/cm^2^)	3	3	3	3
Irradiation time (minutes)	30	30	30	30

**Table 2:** Allocation of human corneas in the treatment groups.

	S-CXL (Group A)	TE-CXL (Group B)	I-CXL 3 mW (Group C)	I-CXL 10 mW (Group D)
Number of corneas	3	3	3	3
Riboflavin	Ricrolin	Ricrolin TE	Ricrolin +	Ricrolin +
Epithelium debridement	YES	NO	NO	NO
Soaking time (minutes)	30	30	10	10
Iontophoresis	NO	NO	YES	YES
Irradiation power (mW/cm^2^)	3	3	30	10
Irradiation time (minutes)	30	30	30	9

**Table 3:** Stress value for 4%, 6%, 8%, and 10% strains in rabbit corneas.

Groups	Stress at 4% (10^3^ Pa)	Stress at 6% (10^3^ Pa)	Stress at 8% (10^3^ Pa)	Stress at 10% (10^3^ Pa)
Group 1				
Untreated	55.7 ± 17.16 (*E* = 1.1 × 10^6^ Pa)	101 ± 37.72 (*E* = 1.8 × 10^6^ Pa)	171.3 ± 73.89 (*E* = 3 × 10^6^ Pa)	270.3 ± 119.1 (*E* = 4.8 × 10^6^ Pa)
Treated	51.3 ± 11.8 (*E* = 1 × 10^6^ Pa)	93.7 ± 29.5 (*E* = 1.7 × 10^6^ Pa)	157.7 ± 59.7 (*E* = 2.7 × 10^6^ Pa)	249.0 ± 100.5 (*E* = 4.3 × 10^6^ Pa)
Group 2				
Untreated	57.3 ± 13.2 (*E* = 1.2 × 10^6^ Pa)	104.7 ± 31.0 (*E* = 1.9 × 10^6^ Pa)	181.0 ± 59.1 (*E* = 3.2 × 10^6^ Pa)	287.7 ± 88.9 (*E* = 5.2 × 10^6^ Pa)
Treated	56.3 ± 6.6 (*E* = 1.1 × 10^6^ Pa)	96.7 ± 12.7 (*E* = 1.7 × 10^6^ Pa)	164.0 ± 27.4 (*E* = 2.8 × 10^6^ Pa)	254.3 ± 37.8 (*E* = 4.5 × 10^6^ Pa)
Group 3				
Untreated	52.7 ± 9.6 (*E* = 1.1 × 10^6^ Pa)	94.7 ± 16.5 (*E* = 1.8 × 10^6^ Pa)	160 ± 25.2 (*E* = 3 × 10^6^ Pa)	260.7 ± 40.5 (*E* = 4.9 × 10^6^ Pa)
Treated	88.3 ± 26.3 (*E* = 2 × 10^6^ Pa)	184.3 ± 75.8 (*E* = 3.6 × 10^6^ Pa)	351.7 ± 171 (*E* = 6.3 × 10^6^ Pa)	603.3 ± 316.7 (*E* = 11.0 × 10^6^ Pa)
Group 4				
Untreated	63.7 ± 14.0 (*E* = 1.1 × 10^6^ Pa)	112.7 ± 22.4 (*E* = 1.8 × 10^6^ Pa)	187.3 ± 31.6 (*E* = 2.8 × 10^6^ Pa)	292.7 ± 45.5 (*E* = 4.4 × 10^6^ Pa)
Treated	51.7 ± 15.3 (*E* = 0.9 × 10^6^ Pa)	87.0 ± 26.9 (*E* = 1.5 × 10^6^ Pa)	134.0 ± 43.3 (*E* = 2.3 × 10^6^ Pa)	201.3 ± 60.2 (*E* = 3.6 × 10^6^ Pa)

Legenda. *E*: calculated Young's modulus.

**Table 4:** Stress value for 4%, 6%, 8%, and 10% strains in human corneas.

Groups	Stress at 4% (10^3^ Pa)	Stress at 6% (10^3^ Pa)	Stress at 8% (10^3^ Pa)	Stress at 10% (10^3^ Pa)
Group A				
S-CXL	194.3 ± 86.7 (*E* = 3.2 × 10^6^ Pa)	349.3 ± 193.8 (*E* = 6 × 10^6^ Pa)	574.3 ± 309.9 (*E* = 11.3 × 10^6^ Pa)	850.3 ± 487.4 (*E* = 21.4 × 10^6^ Pa)
Group B				
TE-CXL	114 ± 32.4 (*E* = 1.8 × 10^6^ Pa)	200.6 ± 50.8 (*E* = 3.1 × 10^6^ Pa)	308.6 ± 58.1 (*E* = 5.2 × 10^6^ Pa)	437.6 ± 53.4 (*E* = 8.6 × 10^6^ Pa)
Group C				
I-CXL 3 mW	123.6 ± 47.5 (*E* = 2.2 × 10^6^ Pa)	238.3 ± 57.2 (*E* = 3.8 × 10^6^ Pa)	388.3 ± 44.8 (*E* = 6.7 × 10^6^ Pa)	576 ± 45 (*E* = 11.8 × 10^6^ Pa)
Group D				
I-CXL 10 mW	150.3 ± 61.6 (*E* = 2.9 × 10^6^ Pa)	276.9 ± 115.4 (*E* = 5.2 × 10^6^ Pa)	449.7 ± 184.4 (*E* = 9.3 × 10^6^ Pa)	661.75 ± 280.9 (*E* = 16.6 × 10^6^ Pa)

Legenda. *E*: calculated Young's modulus.
